# Simultaneous Power Feedback and Maximum Efficiency Point Tracking for Miniaturized RF Wireless Power Transfer Systems

**DOI:** 10.3390/s21062023

**Published:** 2021-03-12

**Authors:** Sebastian Stoecklin, Adnan Yousaf, Gunnar Gidion, Leonhard Reindl, Stefan J. Rupitsch

**Affiliations:** Laboratory for Electrical Instrumentation and Embedded Systems, Department of Microsystems Engineering, University of Freiburg, 79110 Freiburg, Germany; adnan.yousaf@gmail.com (A.Y.); gunnar.gidion@imtek.uni-freiburg.de (G.G.); leonhard.reindl@imtek.uni-freiburg.de (L.R.); stefan.rupitsch@imtek.uni-freiburg.de (S.J.R.)

**Keywords:** wireless power transfer, maximum efficiency point tracking, adaptive impedance matching, power feedback, biomedical systems, wireless microsytems

## Abstract

Near-field interfaces with miniaturized coil systems and low output power levels, such as applied in biomedical sensor systems, can suffer from severe efficiency degradation due to dynamic impedance mismatches, reducing battery life of the power transmitter unit and requiring to increase the level of electromagnetic emission. Moreover, the stability of weakly-coupled power transfer systems is generally limited by transient changes in coil alignment and load power consumption. Hence, a central research question in the domain of wireless power transfer is how to realize an adaptive impedance matching system under the constraints of a simultaneous power feedback to increase the system’s efficiency and stability, while maintaining circuit characteristics such as small size, low power consumption and fast reaction times. This paper presents a novel approach based on a two-stage control loop implemented in the primary-side reader unit, which uses a digital PI controller to maintain the rectifier output voltage for power feedback and an on-top perturb-and-observe controller configuring the setpoint of the voltage controller to maximize efficiency. The paper mathematically analyzes the AC and DC transfer characteristics of a resonant inductive link to design the reactive AC matching network, the digital voltage controller and ultimately the DC-domain impedance matching algorithm. It was found that static reactive L networks result in suitable efficiency levels for coils with sufficiently high quality factor even without adaptive tuning of operational frequency or reactive components. Furthermore, the regulated output voltage of the rectifier is a direct measure of the DC load impedance when using a regular DC/DC converter to supply the load circuits, so that this quantity can be tuned to maximize efficiency. A prototype implementation demonstrates the algorithms in a 40.68 MHz inductive link with load power levels from 10 to 100 mW and tuning time constants of 300 ms, while allowing for a simplified receiver with a footprint smaller than 200 mm^2^ and a self-consumption below 1 mW. Hence, the presented concepts enable adaptive impedance matching with favorable characteristics for low-energy sensor systems, i.e., minimized footprint, power level and reaction time.

## 1. Introduction

The technology of inductive coupling has been widely adopted to supply low-power embedded sensor systems, as it is a reliable and convenient mechanism of wireless power transfer (WPT). Consequently, especially biomedical sensor systems such as cochlear implants to improve hearing [1], retinal implants to reestablish vision and neural implants [2] analyzing brain signals and mitigating diseases such as epilepsy or Parkinson’s disease by electrical stimulation are supplied by this technology [3].

**Figure 1:**
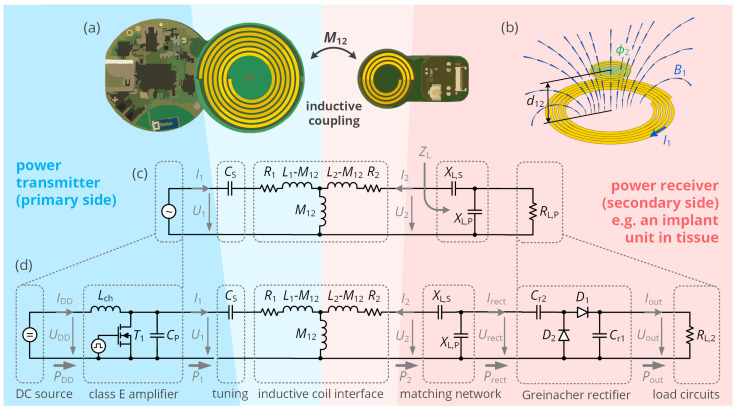
An inductive link for wireless power transfer to a low-power circuit, such as a biomedical implant. (**a**) Physical setup: A primary coil of inductance $L_{1}$ and resistance $R_{1}$ inductively couples to a secondary coil of inductance $L_{2}$ and resistance $R_{2}$ by a mutual inductance $M_{12}$. (**b**) Illustration of the magnetic flux density distribution $B_{1}$ due to the current $I_{1}$ and the magnetic flux $\phi_{2}$ through coil 2. The mutual inductance is defined as $M_{12}:=\phi_{2}/I_{1}$. (**c**) Simple equivalent circuit of the wireless power transfer interface: The inductive coil interface is represented by a T equivalent circuit, the implant AC load resistance $R_{L,P}$ is matched to the secondary coil by a capacitive L matching network. On the primary side, a series capacitance $C_{S}$ (partially) compensates the reactance of $L_{1}$ to allow for increased input power levels. (**d**) Extended equivalent circuit of the wireless power transfer interface: The primary voltage source is replaced by a DC source and a class E amplifier to drive the coil, as well as a Greinacher rectifier (voltage doubler) and a resistive DC load $R_{L,2}$, summarizing voltage converters and the load circuitry itself.

The operational principle of inductive coupling is shown in Figure 1: An alternating primary-side coil current $I_{1}$ generates a magnetic field of flux density $B_{1}$ and therefore a magnetic flux $\phi_{2}$ within a secondary-side coil. This induces a voltage $U_{2}$, which is utilized to charge a secondary battery or to directly supply the load circuits. Particularly motivated by the domain of biomedical engineering, coil systems have been optimized for small size and large power transfer efficiency, which is crucial in order to operate from limited extracorporeal energy storages and minimize heat and electromagnetic emission. Tremendous efforts have been taken to maximize the peak efficiency $\eta_{\max}$ of the corresponding wireless power transfer coils [4,5,6,7,8], optimizing the ratio of primary- and secondary-side real power $P_{i}$ as a target function:

(1) $$\eta_{\max}=\max\left(\frac{P_{2}}{P_{1}}\right)=\frac{\xi_{\max}}{{\left(1+\sqrt{1+\xi_{\max}}\right)}^{2}},$$

where inductive link’s figure of merit $\xi_{\max}$ is composed by the coils’ loss resistances $R_{i}$ and their mutual inductance $M_{12}=\phi_{2}/I_{1}$ [6]

(2) $$\xi_{\max}=\frac{{\left(\omega M_{12}\right)}^{2}}{R_{1}R_{2}}.$$

However, these peak efficiency levels can only be reached for a certain complex load impedance $Z_{L,\mathrm{opt}}$, which also depends on the mutual inductance of the coils, i.e., on their distance and alignment [6,9]:

(3) $$Z_{L,\mathrm{opt}}=\sqrt{\frac{R_{2}}{R_{1}}\left(R_{1}R_{2}+\omega^{2}M_{12}^{2}\right)}-j\omega L_{2}.$$

It is very likely that this optimal impedance does not correspond to the actual load impedance $Z_{L}$ defined by the load circuits. Especially for weakly-coupled systems with extended operational distances and small receiver coils such as used in modern neural implants, efficiency degradations due to these impedance mismatches ($Z_{L,\mathrm{opt}}\left(M_{12}\right)\neq Z_{L}$) impose a great challenge, as they require increased input power levels and, thus, increased electromagnetic emissions and heat as well as reduced battery life of mobile power transmitters. Moreover, wireless power transfer interfaces with weak coupling and parallel-resonant receiver coils represent a power supply with relatively high effective internal resistance, so that fast transient changes in mutual inductance and load power consumption might lead to the interruption of the energy supply [10]. This is particularly crucial for implants with relatively high power demands such as neural implants, which are not feasible to be supplied with rechargeable batteries due to their limited number of recharging cycles and their size. Consequently, transient load changes directly affect the stability of the inductive link and must be compensated as fast as possible.

### 1.1. Preliminary Work

To overcome the limited reliability of the link for weak coupling, mechanisms of *power feedback* have been established in the literature: Here, the supply voltage $U_{\mathrm{DD}}$ or input power of the primary-side amplifier is controlled so that a secondary-side buffer voltage, typically the rectifier output voltage $U_{\mathrm{out}}$, is maintained at a constant level [10,11,12,13]. Changes in coil alignment and load power consumption, which would lead to a significant drop of the buffer voltage, are therefore compensated.

Moreover, changes in coil distance and alignment alter $Z_{L,\mathrm{opt}}$, while changes in load power consumption alter $Z_{L}$, leading to the aforementioned efficiency mismatch. To increase the efficiency as a dynamic reaction to these changes, adaptive impedance matching, also being referred as *maximum efficiency point tracking* (MEPT), has been addressed by various approaches:

As the first category of adaptive matching in the AC domain, being in line with classical RF circuit design, reconfigurable reactive matching networks with switchable arrays of capacitors and/or inductors have been proposed to transform both real and imaginary parts of the load impedance and input impedance to the corresponding optimal values [14,15,16]. However, the complexity of impedance measurement and control on the one hand and the size constraints for low-loss switches and arrays on the other hand limit the applicability to low-power and miniaturized systems.

**Figure 2:**
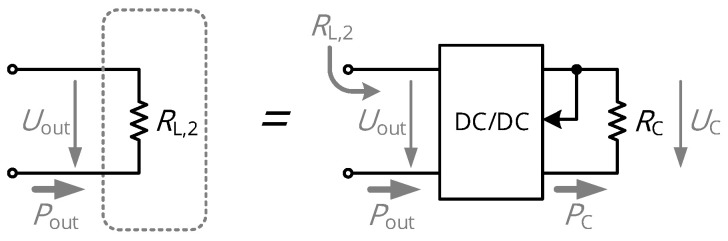
Realization of the DC load resistance $R_{L,2}$ as a combination of an output-controlled DC/DC voltage converter and the load circuits of resistance $R_{C}$. The DC/DC converter thereby acts as an impedance transformer.

Defining the second category of adaptive matching, a voltage conversion in the DC domain is equivalent to a resistance transformation. The concept is classically used in the domain of energy harvesting, i.e., to maximize the power being extracted from solar cells, but has also been applied to the domain of WPT: A DC/DC converter is located at the output of the rectifier stage (see Figure 2) to supply the load circuits with a DC voltage $U_{C}$. Hence, it transforms the DC resistance of the load circuits $R_{C}$ according to the principles of energy conservation, where $P_{C}$ is the power consumed by the load circuits and $\eta_{\mathrm{DC}/\mathrm{DC}}$ is the efficiency of the converter:

(4) $$\begin{array}{cc} P_{C} & =\eta_{\mathrm{DC}/\mathrm{DC}}\cdot P_{\mathrm{out}} \end{array}$$

(5) $$\begin{array}{cc} \frac{U_{C}^{2}}{R_{C}} & =\eta_{\mathrm{DC}/\mathrm{DC}}\cdot \frac{U_{\mathrm{out}}^{2}}{R_{L,2}} \end{array}$$

(6) $$\begin{array}{cc} R_{L,2} & =\eta_{\mathrm{DC}/\mathrm{DC}}\cdot {\left(\frac{U_{\mathrm{out}}}{U_{C}}\right)}^{2}\cdot R_{C}. \end{array}$$

This implies that a DC/DC converter modifies the DC load resistance $R_{L,2}$ seen by the rectification stage, which also changes the resistive input impedance of the rectifier; the imaginary part of the load impedance remains (mostly) unaffected for low frequencies or rectifiers with minimal parasitic capacitances.

In the first circuit and control topologies that applied these principles to the field of WPT, the impedance transformation ratio was determined by the switching duty cycles of individual transistors within the primary- and secondary-side DC/DC converters [17,18]. Efficiency is then maximized by a perturb-and-observe algorithm, which checks the power levels on both transmitter and receiver side, calculates their ratio (i.e., the efficiency), changes the operating point (here, the switching duty cycles) and reiterates until the efficiency is maximized.

As perturbing the duty cycle of several transistors will result in complex control circuits when simultaneously regulating the load voltage, the concept of post-regulation was established: a regular output-controlled DC/DC converter regulates the output voltage of the link, while a primary-side parameter such as the amplifier’s supply voltage [19] or the primary coil’s current [20] are perturbed to optimize efficiency. While this results in a relatively simple circuit topology, the control of the output voltage is not nested with the control of the primary-side amplifier, i.e., no power feedback is established. The resulting systems are therefore operated with long perturbation times to improve stability, which leads to time constants of up to 20 s.

To enable a fast transient settling of the efficiency optimization in the range of 300 ms, one requires control mechanisms maintaining a certain relation of primary-side coil current, load current and coupling factor [21] or a certain transfer function [22,23] across all operational conditions. For example, driving the link with the optimal load resistance for a parallel-resonant link is equivalent to establishing a predetermined ratio of output voltage $U_{2}$ and primary-side coil current $I_{1}$ as proposed by [23], which even allows an intrinsic power feedback. However, the referred approaches are based on a series of particular boundary conditions, such as requiring $\left(\omega^{2}M_{12}^{2}\right)/\left(R_{1}R_{2}\right)\gg 1$, which is not necessarily given for loosely coupled systems with small receiver coils. Moreover, the efficiency of primary-side power amplifier and rectifier can only be included as additional resistive (linear) elements, which is possible for class D amplifiers and active rectifiers applied for operational frequencies in the range of a few 10 to 100 kHz. However, the concepts do not hold for nonlinear class E and Greinacher rectifier stages, which are commonly applied in miniaturized systems with higher operational frequencies (f≥13.56MHz). We published a similar concept based on maintaining a certain voltage transfer function [24]: here, the optimal load resistance $R_{L,2,\mathrm{opt}}$ is determined from a look-up table after the measurement of the mutual inductance $M_{12}$ and correspondingly established by adjusting the rectifier output voltage with an input-controlled DC/DC converter on the secondary side of the link. However, the measurement cycle of $M_{12}$ leads to a momentary interruption of the power supply, a second DC/DC converter stage is required for voltage regulation and power feedback is not intrinsically implemented in this concept.

### 1.2. Outline

As a result, it is the objective of this work to analyze and realize a maximum efficiency point tracker for the application in miniaturized and weakly coupled systems, which optimizes the DC-to-DC power transfer efficiency, implements a power feedback mechanism and requires only a fraction of the low power levels to be transferred, i.e., a few mW. The dynamic optimization should avoid restricting approximations while still providing fast reaction times.

The approach is enabled by the mathematical dimensioning of the reactive matching networks and the generalized efficiency analysis of nonlinear radio-frequency power conditioning circuits in Section 2. From this, the design of a wireless control loop regulating the rectifier output voltage and the design of the maximum efficiency point tracker is derived in Section 3. Finally, Section 4 focuses on the implementation of the concept as a small-sized embedded system, which is characterized with respect to its performance in Section 5. Section 6 finally discusses the results and the system concepts regarding their feasibility and compare them with the state of the art.

Throughout the paper, we examine the impact of the concepts for an exemplary case scenario of a brain implant, such as applied in the context of brain computer interfaces and neural disease research [25]. These systems show a large variability in:

- The separation distance of the power transfer coils $d_{12}$ might change from patient to patient and even within the individual application, as the coils are loosely coupled without alignment structures. We assume a range of $d_{12}$ between 5 and 20 mm.
- The power consumption of the load electronics $P_{C}$ might vary over one decade as a function of resolution and sampling rate (e.g., from 6 to 60 mW for an *Intan RHS2164* [26]) even during operation, as the operating mode of the circuits will adapt to neural activity. Together with microcontroller and data communication, we assume the load power to vary between 25 and 90 mW.

With respect to the electromagnetic interface, the exemplary analysis is based on the capacitively segmented planar spiral coils optimized in [8]. These coils include capacitive series elements within the coil traces to eliminate parasitic currents in the stray capacitances of adjacent coil traces and to avoid phase shifts of the current along the conductor, so that the coils feature a high maximum efficiency even in the environment of lossy dielectric media. The coil dimensions and equivalent circuit parameters are provided in Table 1.

## 2. Theoretical Efficiency Analysis

In the first part of our analysis, we focus on the efficiency characteristics of the unregulated link including primary-side power amplifier and rectifier such as shown in Figure 1. To simplify the expressions throughout the mathematical derivations of the following section, we introduce the impedance matrix relating the input voltages and currents of the inductive link, which defines the reactances $X_{1}$, $X_{2}$ and $X_{12}$:

(7) $$\left(\begin{array}{c} U_{1}\\ U_{2} \end{array}\right)=\left(\begin{array}{cc} R_{1}+jX_{1} & jX_{12}\\ jX_{12} & R_{2}+jX_{2} \end{array}\right)\cdot \left(\begin{array}{c} I_{1}\\ I_{2} \end{array}\right)=\left(\begin{array}{cc} R_{1}+j\omega L_{1}+\frac{1}{j\omega C_{S}} & j\omega M_{12}\\ j\omega M_{12} & R_{2}+j\omega L_{2} \end{array}\right)\cdot \left(\begin{array}{c} I_{1}\\ I_{2} \end{array}\right).$$

### 2.1. Reactive Matching Networks

The optimal load impedance $Z_{L,\mathrm{opt}}$ of (3) already indicates that the load must provide an imaginary part that cancels the reactance $\omega L_{2}$ of the secondary coil. In the literature, this reactance is provided either by a series capacitor for rather high-power systems or by a parallel capacitor for low-power systems. Hybrid approaches are also considered [27], but not generically optimized showing the full impact of the design. Hence, we take the hybrid approach of a capacitive L network, such as shown in Figure 1c, and make it subject to a straightforward mathematical analysis. First, we define that the series reactance $X_{L,S}$ shall compensate a fraction α of the secondary coil’s reactance, so that

(8) $$\begin{array}{c} X_{L,S}=-\alpha X_{2}=-\alpha \omega L_{2}. \end{array}$$

Then, the overall impedance resulting from the AC load resistance $R_{L,P}$, the parallel reactance $X_{L,P}$ and the series reactance $X_{L,S}$ shall be equal to the optimal load impedance of (3):

(9) $$\begin{array}{c} Z_{L,\mathrm{opt}}\overset{!}{=}jX_{L,S}+\frac{jX_{L,P}\cdot R_{L,P}}{jX_{L,P}+R_{L,P}}. \end{array}$$

Using (9) and solving for the parallel components, we obtain:

(10) $$\begin{array}{cc} R_{L,P,\mathrm{opt}}\left(X_{12}\right) & =\frac{{\left(1-\alpha \right)}^{2}X_{2}^{2}R_{1}+R_{1}R_{2}^{2}+R_{2}X_{12}^{2}}{\sqrt{R_{1}R_{2}\left(R_{1}R_{2}+X_{12}^{2}\right)}}, \end{array}$$

(11) $$\begin{array}{cc} X_{L,P,\mathrm{opt}}\left(X_{12}\right) & =-\left(\left(1-\alpha \right)X_{2}+\frac{R_{1}R_{2}^{2}+R_{2}X_{12}^{2}}{\left(1-\alpha \right)R_{1}X_{2}}\right). \end{array}$$

These generic results automatically specify the optimal values for the particular cases of series resonance (α=1, where $X_{L,P,\mathrm{opt}}\to \infty$) and parallel resonance (α=0, where $X_{L,S}=0$). It can be noted that the optimal load resistance $R_{L,P,\mathrm{opt}}$ is a function of $X_{12}$ for every α, again proving the initial request for adaptive impedance matching. Moreover, the parallel reactance is also a function of $X_{12}$ for α≠1, which would require an adaptive compensation. The tuning of the reactance is, however, associated with additional complexity and footprint, such as imposed by capacitor arrays or varactor diodes with high supply voltages. Furthermore, the alternative approach of tuning the operational frequency, as suggested by Huang et al. [23], is not feasible for radio frequency systems, which are constrained by regulated ISM frequency bands, e.g., at 13.56 MHz or 40.68 MHz.

As neither frequency tracking nor variable capacitances are feasible in a low-power, small-size and radio-frequency implementation, let us assume a static reactance $X_{L,P}$ in the matching network. Then, the efficiency $\eta_{\mathrm{link}}$ can be computed from linear circuit analysis to be

(12) ηlink=Re(−U2I2*)Re(U1I1*)=RL,PX122XL,P2g(X12,RL,P,XL,P),

where ${}^{*}$ denotes the complex conjugate and

(13) $$\begin{array}{cc} g(X_{12},R_{L,P},X_{L,P})= & R_{1}[R_{2}^{2}\left(R_{L,P}^{2}+X_{L,P}^{2}\right)+2R_{2}R_{L,P}X_{L,P}^{2}+\\ \; & \ldots +R_{L,P}^{2}{\left(-\alpha X_{2}+X_{2}+X_{L,P}\right)}^{2}+{\left(\alpha -1\right)}^{2}X_{2}^{2}X_{L,P}^{2}]+\\ \; & \ldots +X_{12}^{2}\left[R_{2}\left(R_{L,P}^{2}+X_{L,P}^{2}\right)+R_{L,P}X_{L,P}^{2}\right]. \end{array}$$

This expression can be maximized by demanding $\partial \eta_{\mathrm{link}}/\partial R_{L,P}=0$ and by solving the result for $R_{L,P}$, which yields the load resistance $R_{L,P,\mathrm{st},\mathrm{opt}}$ maximizing efficiency for a static matching network

(14) $$\begin{array}{cc} R_{L,P,\mathrm{st},\mathrm{opt}} & =\frac{|X_{L,P}|\sqrt{R_{1}\left(R_{2}^{2}+{\left(\alpha -1\right)}^{2}X_{2}^{2}\right)+R_{2}X_{12}^{2}}}{\sqrt{R_{1}\left(R_{2}^{2}+{\left(-\alpha X_{2}+X_{2}+X_{L,P}\right)}^{2}\right)+R_{2}X_{12}^{2}}}. \end{array}$$

While this determines the load resistance for a particular mutual impedance $X_{12}$, it is still unknown which parallel reactance $X_{L,P}$ to choose. We can now match the link for a nominal mutual impedance $X_{12}^{m}$ according to (11) and then derive its efficiency level at a different mutual impedance $X_{12}$:

(15) $$\begin{array}{c} \eta_{\mathrm{link}}\left(X_{12}|X_{12}^{m}\right)=\eta_{\mathrm{link}}\left(X_{12}\right){|}_{X_{L,P}=X_{L,P,\mathrm{opt}}\left(X_{12}^{m}\right)}. \end{array}$$

This basically means that we statically tune the link for a particular coil distance and alignment (characterized by $X_{12}^{m}$) and then change the coil distance to yield $X_{12}$ and a reduced efficiency $\eta_{\mathrm{link}}\left(X_{12}|X_{12}^{m}\right)<\eta_{\max}\left(X_{12}\right)$.

Given that the maximum mutual impedance between the coils is $X_{\max}=\sqrt{X_{1}X_{2}}$ (corresponding to a coupling factor of 1), a worst-case efficiency degradation from the optimal efficiency can occur if:

1. the load is matched for strong coupling, but operates at very weak coupling ($X_{12}=0,X_{12}^{m}=X_{\max}$); or
2. the load is matched for very weak coupling, but operates at strong coupling ($X_{12}=X_{\max},X_{12}^{m}=0$).

The efficiency degradation is expressed best as a normalized efficiency, being the ratio of the resulting link efficiency and the maximum link efficiency, i.e., $r_{1}=\eta_{\mathrm{link}}\left(0|X_{\max}\right)/\eta_{\max}\left(0\right)$ for the first and $r_{2}=\eta_{\mathrm{link}}\left(X_{\max}|0\right)/\eta_{\max}\left(X_{\max}\right)$ for the second scenario. Here, a higher ratio is better and r=1 means that no degradation is present. Substituting the coil reactances $X_{i}=Q_{i}\cdot R_{i}$ by introducing the coil quality factor $Q_{i}$ allows expressing this normalized efficiency for both worst-case scenarios as pure functions of the compensation ratio α and the coil quality factors $Q_{i}$.

**Figure 3:**
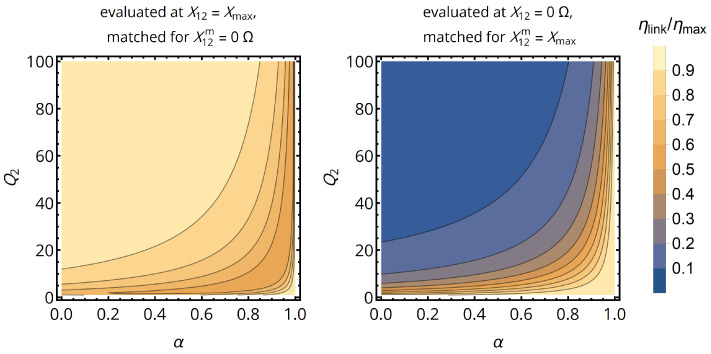
Normalized efficiency as a metric of performance degradation when matching the coil system for a nominal mutual impedance $X_{12}^{m}$, but operating it at a different configuration $X_{12}$. Matching the system for a small nominal mutual impedance does barely reduce efficiency for α<0.8 and higher values of $Q_{2}$. For all graphs, we assumed $Q_{1}=100$.

This is shown in Figure 3 for both worst case scenarios and a primary coil with $Q_{1}=100$; the secondary coil’s quality factor $Q_{2}$ and the compensation ratio α are the variables. In the case of a secondary coil with sufficiently high quality factor (e.g., $Q_{2}>25$ for α=0.5), the optimization of the matching network for low coupling ($X_{12}^{m}=0$) results in worst-case efficiency levels, which are within 90% of the maximum efficiency. Consequently, there is practically no need for adaptive compensation of the secondary-side parallel capacitor or the operational frequency for high-quality-factor coils, as the static impedance matching network will perform almost equally well when being matched for a small $X_{12}^{m}$. As we did not use any approximations to obtain this result, it holds regardless of coil size and frequency. In summary, the optimal parallel load reactance and resistance for static matching (index sm) are given by

(16) $$\begin{array}{cc} \; & X_{L,P,\mathrm{opt},\mathrm{sm}}={\left.X_{L,P,\mathrm{opt}}\right|}_{X_{12}\to 0}\overset{\left(11\right)}{=}-\left(\left(1-\alpha \right)X_{2}+\frac{R_{2}^{2}}{\left(1-\alpha \right)X_{2}}\right), \end{array}$$

(17) $$\begin{array}{cc} \; & R_{L,P,\mathrm{opt},\mathrm{sm}}\overset{\left(14)+(16\right)}{=}\sqrt{\frac{{\left(R_{2}^{2}+{\left(\alpha -1\right)}^{2}X_{2}^{2}\right)}^{2}\left(R_{1}\left(R_{2}^{2}+{\left(\alpha -1\right)}^{2}X_{2}^{2}\right)+R_{2}X_{12}^{2}\right)}{R_{2}\left(R_{1}R_{2}\left(R_{2}^{2}+{\left(\alpha -1\right)}^{2}X_{2}^{2}\right)+{\left(\alpha -1\right)}^{2}X_{12}^{2}X_{2}^{2}\right)}}. \end{array}$$

Therefore, designing the matching network means selecting the only residual variable, namely the compensation ratio α. As generally considered in the literature, a series-resonant tuning (α=1) at the receiver is preferred for high power levels, while a parallel-resonant tuning (α=0) is applied to low-power systems. As shown in the following, this also applies here, but with the option to specifically adapt the matching to the required power levels by the hybrid approach: First, it must be noted that the rectifier’s input voltage range $\left[{\hat{U}}_{\mathrm{rect},\min},{\hat{U}}_{\mathrm{rect},\max}\right]$ is limited by the characteristics of rectifier and subsequent voltage regulator. For example, for a Greinacher rectifier acting as a voltage doubler and a modern buck-boost regulator supporting an input voltage between 2 and 20 V, the voltage $U_{\mathrm{rect}}$ might range between 1 and 10 V. When applying the optimal resistive load according to (17) in the case of optimal matching (we address how to achieve this below), the voltage range will directly bound the receivable power range of the WPT interface:

(18) $$\begin{array}{c} P_{\min}=\frac{{\hat{U}}_{\mathrm{rect},\min}^{2}}{2R_{L,P,\mathrm{opt},\mathrm{sm}}},P_{\max}=\frac{{\hat{U}}_{\mathrm{rect},\max}^{2}}{2R_{L,P,\mathrm{opt},\mathrm{sm}}}. \end{array}$$

**Figure 4:**
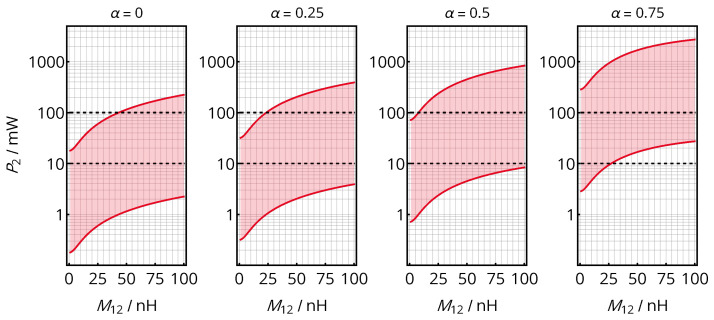
Power bands in which an optimal matching to the load $R_{L,2,\mathrm{opt},\mathrm{SM}}\left(M_{12}\right)$ is possible for an limited voltage range $\left[{\hat{U}}_{\mathrm{rect},\min},{\hat{U}}_{\mathrm{rect},\max}\right]$ (here, [1V,10V]). The compensation ratio α allows adapting the power band to the needs of the load circuits.

As $R_{L,P,\mathrm{opt},\mathrm{sm}}$ is a function of $X_{12}$, the result is a $M_{12}$-dependent power band to be realized for the optimal matching, shown in Figure 4 for the given coil system. The graph indicates that certain power levels might not be achieved with optimal matching for all coupling situations and that α can be selected to tailor the power band for the target consumption of the load device. In the exemplary implementation of this work, α=0.5 is therefore used to successfully match power levels from 10 to 100 mW (see Section 1.2) for any suitable mutual inductance ($10\;\mathrm{nH}<M_{12}<80\;\mathrm{nH}$, cf. Table 1). With (9) and (16), this results in $X_{L,S}\approx X_{L,P}\approx -24.3\;\Omega$ and thus in $C_{L,S}\approx C_{L,P}\approx 160\;\mathrm{pF}$.

### 2.2. Coil Driver and Rectification Stages

While the previous section focuses on the coil interface and the corresponding matching network, the actual WPT interface also includes the additional components shown in Figure 1d: On the primary side, a coil driver circuit converts a DC supply voltage $U_{\mathrm{DD}}$ into the required AC input voltage $U_{1}$. A commonly applied circuit topology is the class E amplifier, which is known for its high drain efficiency (theoretically up to 100%) as well as its load dependence and efficiency degradation with a variable load resistance [28]. On the secondary side, a Greinacher rectifier, as a commonly applied RF circuit [29], handles the conversion from the AC voltage level $U_{\mathrm{rect}}$ to the DC voltage level $U_{\mathrm{out}}$. It is inherent to both of these circuits that they act as nonlinear power transfer stages, which also implies individual power transfer efficiency levels that scale with voltage levels and load impedances. As simplified equivalent circuit models [30] do not fully account for the nonlinear behavior and for the parasitic capacitances of the components also influencing the power transfer characteristics, the harmonic balance method as a state-of-the-art RF circuit simulation algorithm shall be used to characterize the system. Here, the DC, the fundamental frequency and the harmonic components of every nodal voltage and branch current can be obtained for a given set of DC input voltage $U_{\mathrm{DD}}$, mutual inductance $M_{12}$ and DC load resistance $R_{L,2}$. From these spectral components, the steady-state and time-domain voltages $u_{n}\left(t\right)$ and currents $i_{n}\left(t\right)$ can be extracted by an inverse Fourier transform, which allows computing the corresponding effective input power levels

(19) $$\begin{array}{cc} P_{n} & =\frac{1}{T}\int_{0}^{T}u_{n}\left(t\right)\cdot i_{n}\left(t\right)dt, \end{array}$$

and, therefore, the efficiency levels of the power amplifier $\eta_{\mathrm{amp}}$, the inductive link including matching network $\eta_{\mathrm{link}}$, the rectifier $\eta_{\mathrm{rect}}$ and the overall system $\eta_{\mathrm{total}}$:

(20) $$\begin{array}{c} \eta_{\mathrm{amp}}=\frac{P_{1}}{P_{\mathrm{DD}}},\eta_{\mathrm{link}}=\frac{P_{\mathrm{rect}}}{P_{1}},\eta_{\mathrm{rect}}=\frac{P_{\mathrm{out}}}{P_{\mathrm{rect}}},\eta_{\mathrm{total}}=\frac{P_{\mathrm{out}}}{P_{\mathrm{DD}}}. \end{array}$$

**Figure 5:**
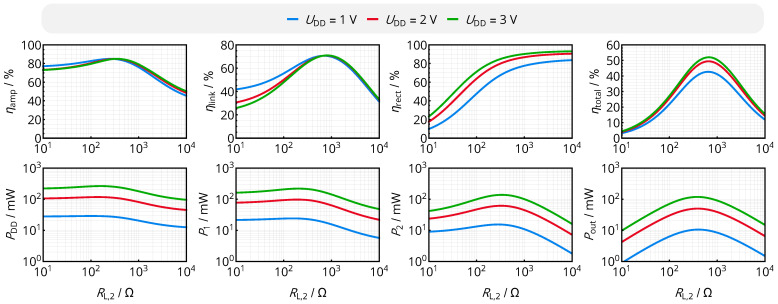
Power and efficiency levels of the main components of the WPT interface versus the DC load resistance $R_{L,2}$. All components’ efficiency levels scale differently with load resistance and different overall power levels determined by $U_{\mathrm{DD}}$. The overall efficiency $\eta_{\mathrm{total}}$ is still optimized by a dedicated DC load resistance $R_{L,2}$.

Evaluating these expressions for the exemplary coil system of Table 1 and the configuration of the electronic components of Table 2 results in the power and efficiency curves in Figure 5. The harmonic balance algorithm was executed using *Keysight Advanced Design System 2016* with the corresponding SPICE models of the nonlinear devices. From the results, several conclusions can be drawn:

- The rectifier efficiency strongly scales with the input voltage level (defined by the primary-side supply voltage $U_{\mathrm{DD}}$) and the load resistance $R_{L,2}$. As increasing both quantities increases the ratio of output voltage and diode voltage drop due to increased overall voltage levels for $U_{\mathrm{DD}}$ and reduced diode currents for $R_{L,2}$, efficiency $\eta_{\mathrm{rect}}$ is also increased. Rectifier output voltages being far larger than the diode voltage drops (below the breakdown voltage) and high load resistances are thus the key to optimize the RF rectification stages.
- The rectifier acts as an impedance transformer. From the power balance at the input and output of the rectifier, the following relation of AC input resistance $R_{L,P}$ and DC load resistance $R_{L,2}$ can be derived:
  (21) $$\begin{array}{cc} \; & \eta_{\mathrm{rect}}\cdot \frac{U_{\mathrm{rect}}^{2}}{2R_{L,P}}=\frac{U_{\mathrm{out}}^{2}}{R_{L,2}}, \end{array}$$
  (22) $$\begin{array}{cc} \Rightarrow & R_{L,P}=\frac{R_{L,2}}{2}\cdot \eta_{\mathrm{rect}}\cdot {\left(\frac{U_{\mathrm{rect}}}{U_{\mathrm{out}}}\right)}^{2}. \end{array}$$
  For an ideal voltage doubler, i.e., $\eta_{\mathrm{rect}}\approx 1$ and $U_{\mathrm{out}}\approx 2U_{\mathrm{rect}}$, we obtain $R_{L,P}\approx 1/8R_{L,2}$. Given that the optimal AC load resistance is approximately 120 Ω according to (17), the DC load resistance to optimize the (isolated) link efficiency is approximately 960 Ω, which qualitatively matches the optimum of $\eta_{\mathrm{link}}$ in Figure 5.
- Changing the load resistance directly modifies the input impedance for a non-zero inductive coupling. As a result, the class E amplifier’s drain efficiency varies with $R_{L,2}$.
- The output power $P_{\mathrm{out}}$ strongly scales with $R_{L,2}$. As there is a dedicated power maximum for a given input voltage $U_{\mathrm{DD}}$ (which is not falling together with the maximum of the total efficiency), this input voltage must be adaptively scaled to fulfill the power needs of the load circuits.
- In summary, power amplifier and rectifier show a strong impact on the overall efficiency: they reduce the maximum total efficiency and modify the relation of overall efficiency and load resistance. It is noteworthy that the load optimizing link efficiency and the load optimizing overall efficiency do not necessarily fall together. As a result, the power electronics should be incorporated into any adaptive efficiency-optimization mechanism.

## 3. Adaptive Power-Conditioning Circuit Concepts

**Figure 6:**
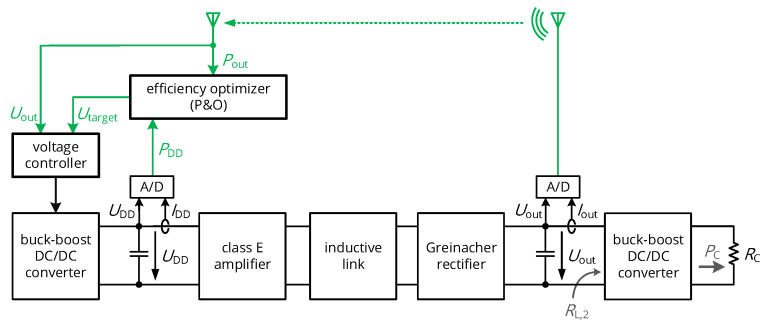
Circuit topology for simultaneous power feedback and maximum efficiency point tracking: A digital primary-side controller regulates the rectifier’s output voltage to stabilize the wirelessly supplied system even in case of load and coil alignment transients. On top of this voltage controller for power feedback, a perturb-and-observe controller varies the setpoint on the voltage controller $U_{\mathrm{target}}$ to tune the effective DC load resistance $R_{L,2}$ and optimize the overall system efficiency. Efficiency data for the perturb-and-observe controller are acquired by the measurement of the DC power levels on primary and secondary side.

After analyzing the analog core circuits of the WPT interface, we deduce a control topology to always provide the required load power and to adaptively operate the interface with the load resistance that results in a maximized overall efficiency. This is allowed by the circuit topology shown in Figure 6, which is explained in detail in the following sections. 

### 3.1. Power Feedback

As mentioned in the state-of-the-art section, power feedback can be established by the control of the rectifier’s output voltage $U_{\mathrm{out}}$. This involves the measurement of this quantity on the secondary side, which is subsequently transmitted over a wireless link to the primary side, where a digital controller modifies the input voltage $U_{\mathrm{DD}}$ by a configurable DC/DC converter to regulate $U_{\mathrm{out}}$. Two design steps are required to realize this system: the identification of the transfer function $G\left(s\right)=U_{\mathrm{out}}\left(s\right)/U_{\mathrm{DD}}\left(s\right)$ and the appropriate dimensioning on the controller’s z-domain transfer function C[z]. In the following derivation, the charging behavior of the buffering capacitor $C_{r1}$ at the rectifier’s output is assumed to be dominant, so that the transient behavior of the RF circuits operated at 40.68 MHz is considered negligible.

**Figure 7:**
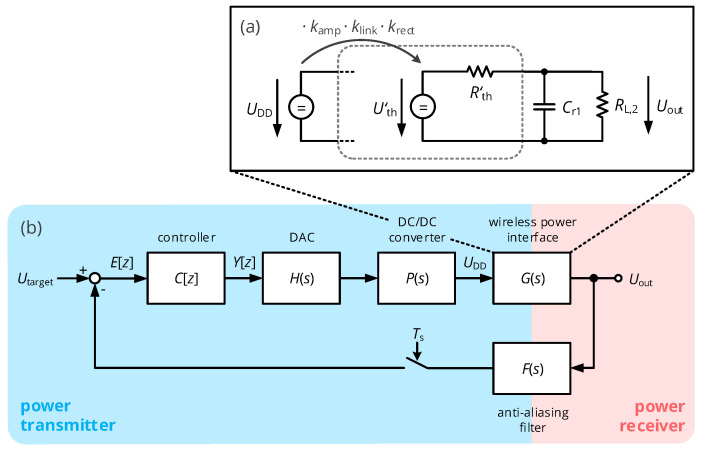
(**a**) DC-domain Thévenin equivalent circuit of the complete wireless power transfer link including class E amplifier, coil interface and rectifier into a simple PT1 element. (**b**) Voltage control loop of the wireless power transfer interface: The interfaces output voltage $U_{\mathrm{out}}$ is digitized and provided to a digital controller, which interfaces a primary-side DC/DC converter through a digital-to-analog converter to establish the target voltage $U_{\mathrm{target}}$.

To relate $U_{\mathrm{DD}}$ and $U_{\mathrm{out}}$ in a compact and comprehensible way, we assume that both the class E amplifier and the rectifier act as a voltage converter with constant conversion ratio. For the class E converter, the ratio of $k_{\mathrm{amp}}=U_{1}/U_{\mathrm{DD}}$ was determined by circuit simulation, where the class E amplifier’s gain yielded $k_{\mathrm{amp}}\approx 1.4$ for the given circuit configuration. The rectifier’s conversion ratio is assumed to be $k_{\mathrm{rect}}\approx 2$, in line with its voltage-doubling properties. Given those boundary conditions, we generate DC Thévenin equivalent circuit for the complete chain of power amplifier, coil interface, matching network and rectifier, such as shown in Figure 7a: First, the voltage ratio of the AC amplitudes ${\hat{U}}_{1}$ and ${\hat{U}}_{\mathrm{rect}}$ can be obtained from the analysis of the circuit of Figure 1c when considering $R_{L,P}\approx R_{L,2}/\left(2k_{\mathrm{rect}}^{2}\right)$, which results in

(23) U^rect≈U^1·klink·RL,22(RL,2+2krect2·Re(Zth))2+(2krect2·Im(Zth))2.

Applying the voltage gains $k_{\mathrm{amp}}$ and $k_{\mathrm{rect}}$, we obtain

(24) Uout=UDD·kamp·krect·klink·RL,22(RL,2+2krect2·Re(Zth))2+(2krect2·Im(Zth))2,

where

(25) $$\begin{array}{cc} k_{\mathrm{link}} & =\left|\frac{X_{12}X_{L,P}}{X_{12}^{2}+\left(R_{1}+jX_{1}\right)\left(R_{2}+j\left(X_{2}+X_{L,P}+X_{L,S}\right)\right)}\right|, \end{array}$$

(26) $$\begin{array}{cc} Z_{\mathrm{th}} & =\frac{jX_{L,P}\left(X_{12}^{2}+\left(R_{1}+jX_{1}\right)\left(R_{2}+j\left(X_{2}+X_{L,S}\right)\right)\right)}{X_{12}^{2}+\left(R_{1}+jX_{1}\right)\left(R_{2}+j\left(X_{2}+X_{L,P}+X_{L,S}\right)\right)}. \end{array}$$

This can then be compared with the output voltage of the DC Thévenin equivalent circuit of Figure 7a

(27) $$\begin{array}{c} U_{\mathrm{out}}=U_{\mathrm{th}}^{\prime }\cdot \frac{R_{L,2}}{R_{L,2}+R_{\mathrm{th}}^{\prime }}, \end{array}$$

to yield the equivalent-circuit parameters

(28) $$\begin{array}{cc} U_{\mathrm{th}}^{\prime } & =U_{\mathrm{DD}}\cdot k_{\mathrm{amp}}\cdot k_{\mathrm{rect}}\cdot k_{\mathrm{link}}, \end{array}$$

(29) Rth′=(RL,2+2krect2·Re(Zth))2+(2krect2·Im(Zth))2−RL,2.

As this DC Thévenin equivalent circuit sees the large buffering capacitor $C_{r1}$ of the rectifier at its output, the system’s transient behavior is determined by the circuit consisting of $R_{\mathrm{th}}^{\prime }$, $C_{r1}$ and $R_{L,2}$, which leads to a PT1 system with the transfer function

(30) $$\begin{array}{c} G\left(s\right)=\frac{k_{\mathrm{WPT}}}{1+\tau_{\mathrm{WPT}}s}=\frac{k_{\mathrm{rect}}\cdot k_{\mathrm{link}}\cdot k_{\mathrm{amp}}\cdot \frac{R_{L,2}}{R_{L,2}+R_{\mathrm{th}}^{\prime }}}{1+\left(\frac{C_{r1}R_{L,2}R_{\mathrm{th}}^{\prime }}{R_{L,2}+R_{\mathrm{th}}^{\prime }}\right)s}, \end{array}$$

where *s* is the complex variable of the Laplace transform.

As a result, (30) summarizes the DC-source-to-DC-load transfer function, in which gain and time constant are functions of the mutual coil coupling ($k_{\mathrm{link}}$ and $R_{\mathrm{th}}^{\prime }$ scale with $X_{12}$) and the load resistance $R_{L,2}$. Hence, the complete analog chain, consisting of the blocks illustrated in Figure 7b, can be characterized by the transfer function

(31) $$\begin{array}{ccccc} G_{a}\left(s\right) & =H(s) & \cdot P(s) & \cdot G(s) & \cdot F(s) \end{array}$$

(32) $$\begin{array}{ccccc} \; & =\left(\frac{1-e^{-sT_{s}}}{sT_{s}}\right) & \cdot \left(\frac{1}{1+\tau_{\mathrm{PS}}s}\right) & \cdot \left(\frac{k_{\mathrm{WPT}}}{1+\tau_{\mathrm{WPT}}s}\right) & \cdot \left(\frac{1}{1+\tau_{\mathrm{AA}}s}\right), \end{array}$$

with the sampling time $T_{s}=1\;\mathrm{ms}$ and the experimentally determined time constants of the DC/DC converter $\tau_{\mathrm{PS}}=100\;µs$ and of the anti-aliasing filter $\tau_{\mathrm{AA}}=300\;µs$.

For the control of the system, a digital PID controller with the z-domain transfer function of [31] (p. 190)

(33) $$\begin{array}{c} C\left[z\right]=\frac{\left(2K_{P}T_{I}T_{S}+4T_{I}T_{D}+T_{S}^{2}\right)+\left(2T_{S}^{2}-8T_{I}T_{D}\right)z^{-1}+\left(T_{S}^{2}+4T_{I}T_{D}-2K_{P}T_{I}T_{S}\right)z^{-2}}{2T_{I}T_{S}\left(1-z^{-1}\right)} \end{array}$$

is considered, which results in the following update equation for the controller’s output Y[n] as a function of the error term $E\left[n\right]=(U_{\mathrm{target}}\left[n\right]-U_{\mathrm{out}}\left[n\right])$:

(34) $$\begin{array}{cc} Y[n]= & Y\left[n-1\right]+\frac{2K_{P}T_{I}T_{S}+4T_{I}T_{D}+T_{S}^{2}}{2T_{I}T_{S}}\cdot E\left[n\right]+\cdots \\ \; & +\frac{2T_{S}^{2}-8T_{I}T_{D}}{2T_{I}T_{S}}\cdot E\left[n-1\right]+\frac{T_{S}^{2}+4T_{I}T_{D}-2K_{P}T_{I}T_{S}}{2T_{I}T_{S}}\cdot E\left[n-2\right] \end{array}$$

With the z-domain transfer function $G_{a}\left[z\right]$ as the discrete-time equivalent of $G_{a}\left(s\right)$, the transfer function of the open loop is hence given by

(35) $$\begin{array}{c} G_{o}\left[z\right]=C\left[z\right]\cdot G_{a}\left[z\right], \end{array}$$

which allows determining the crossover frequency $f_{0}$ as a measure of settling time and the phase margin $\varphi_{\mathrm{pm}}$ as a measure of system stability by the following equations for every possible mutual inductance and load resistance:

(36) $$\begin{array}{cc} \; & |G_{o}\left(e^{j2\pi f_{0}}\right)|=1, \end{array}$$

(37) $$\begin{array}{cc} \; & \varphi_{\mathrm{pm}}=180{}^{\circ}+\arg\left(G_{o}\left(e^{j2\pi f_{0}}\right)\right). \end{array}$$

**Figure 8:**
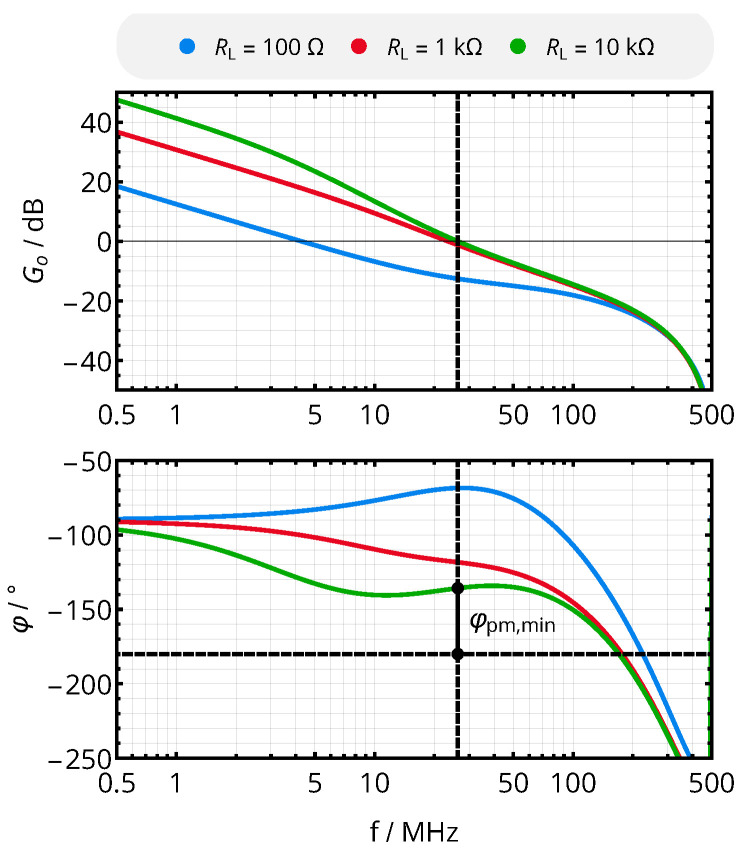
Bode plot of the open-loop transfer function $G_{o}\left[e^{j2\pi \;f_{0}}\right]$ for $M_{12}=10\;\mathrm{nH}$, the control parameters $\left(K_{P},T_{I},T_{D}\right)=\left(0.7,0.01\;s,0\;s\right)$ and different DC load resistances $R_{L,2}$, resulting in the minimal phase margin of 44° for $R_{L,2}=10\;k\Omega$.

Now, every set of the controller’s parameters (DC gain $K_{P}$, integral time $T_{I}$ and derivative time $T_{D}$) can be analyzed for its minimal crossover frequency and phase margin across the range of feasible system parameters, i.e., for a mutual inductance between 10 and 80 nH and a load resistance between 100 and 10,000 Ω. For the given system, the control parameters $\left(K_{P},T_{I},,T_{D}\right)=\left(0.7,0.01\;s,0\;s\right)$ resulted in a favorable system with a minimal phase margin of $\varphi_{\mathrm{pm},\min}=44{}^{\circ}$ for $M_{12}=10\;\mathrm{nH}$ and $R_{L,2}=10\;k\Omega$, thus being stable across all analyzed operating points. The corresponding Bode plot of the open-loop transfer function is shown in Figure 8; its variation with the load resistance $R_{L,2}$ indicates the dependence of the link’s transfer function on the operating point. By setting $T_{D}=0\;s$, we omit the derivative term to obtain a PI controller.

### 3.2. Maximum Efficiency Point Tracking

Having assured that the load is always supplied with the required power level $P_{C}$ due to a stable control of the rectifier’s output voltage, the efficiency optimization is the ultimate challenge to be solved. Although regulated by the given feedback loop, $U_{\mathrm{out}}$ should not be directly utilized to supply the load circuits, as it is undefined during startup and as it might not suppress under- and overshoots stemming from fast load transients. Hence, a natural way to supply the load circuits with a constant voltage independent of the rectifier’s output voltage is to insert an output-regulated DC/DC converter after the rectification stage, favorably a buck-boost converter with a wide input range below and above the load circuit’s supply voltage. These devices are recently available with a high converter efficient $\eta_{\mathrm{DC}/\mathrm{DC}}$, and the power balance from Equation (4) can be rewritten as

(38) $$\begin{array}{c} R_{L,2}=\eta_{\mathrm{DC}/\mathrm{DC}}\cdot \frac{U_{\mathrm{out}}^{2}}{P_{C}}. \end{array}$$

This means that the link’s DC load resistance $R_{L,2}$, represented by the DC/DC converter and the load circuits, is a direct and monotonic function of the rectifier’s output voltage $U_{\mathrm{out}}$, which is the voltage we control for power feedback. Hence, the setpoint of this voltage, given by $U_{\mathrm{target}}$, defines the load resistance and can therefore be tuned to optimize the link efficiency. This does not affect the circuit’s supply voltage $U_{C}$, which is decoupled from $U_{\mathrm{out}}$ by the buck-boost converter. Hence, an additional control layer defines the setpoint $U_{\mathrm{target}}$ of the voltage control loop, such as shown in Figure 6. The method of tuning to a new load impedance by defining the setpoint of the rectifier’s output voltage is the unique feature of this system compared to the state of the art, and it is particularly beneficial due to the simultaneous power feedback being established.

A method for modifying the control variable (here, $U_{\mathrm{target}}$) to establish the optimal load impedance and maximize efficiency is the *perturb-and-observe* algorithm, which is massively applied in the field of energy harvesting for the purpose of maximum power point tracking (see, e.g., [32]) and has also been applied in several WPT systems referred to in the state-of-the-art section (e.g., [17,18,20]). Its basic idea is to increase or decrease the control variable by a certain amount (*perturbation*) in the first step and then to measure the effect on the target metric (*observation*). If the latter has changed favorably, the perturbation will be increased into the same direction; otherwise, the direction will be changed. In the presented application, this means that the setpoint of the rectifier’s output voltage $U_{\mathrm{target}}$ is increased by a perturbation step $\Delta U_{\mathrm{MEPT}}$. Then, input and output power of the link are measured and transferred to the primary-side control interface in order to compute the momentary efficiency. If the efficiency has increased, $U_{\mathrm{target}}$ will be further increased in the next step, and reduced otherwise:

(39) $$\begin{array}{cc} \Delta U_{\mathrm{MEPT}}\left[k\right] & =\left\{\begin{array}{cc} \Delta U_{\mathrm{MEPT}}\left[k-1\right] & \mathrm{if}\;\eta_{\mathrm{WPT}}\left[k\right]>\eta_{\mathrm{WPT}}\left[k-1\right],\\ -\Delta U_{\mathrm{MEPT}}\left[k-1\right] & \mathrm{otherwise}. \end{array}\right. \end{array}$$

(40) $$\begin{array}{cc} U_{\mathrm{target}}\left[k\right] & =U_{\mathrm{target}}\left[k-1\right]+\Delta U_{\mathrm{MEPT}}\left[k\right]. \end{array}$$

There are questions that still remain: When should the next perturbation step start? How large should the perturbation be? A custom approach of the proposed tracking scheme is to *start the next perturbation step* when the error of the voltage controller $\left|E\left[n\right]|=\right|U_{\mathrm{target}}\left[n\right]-U_{\mathrm{out}}\left[n\right]|$ is sufficiently low, which means that no load or coupling transients have recently occurred (a situation in which a perturbation of the target voltage might reduce stability) and that the rectifier voltage has settled, so that a stable efficiency measurement can be performed. The *size of the perturbation step* has to be adapted to the standard deviation of the efficiency measurement: if the standard deviation is high, there will be a certain probability of detecting the wrong sign of an efficiency change and of stepping into the false search direction. On the one hand, a higher perturbation step allows for a more distinct change in efficiency and enables a faster convergence towards the optimum. On the other hand, the optimum cannot be precisely reached. Hence, speed and precision can be traded upon the needs of the application.

**Figure 9:**
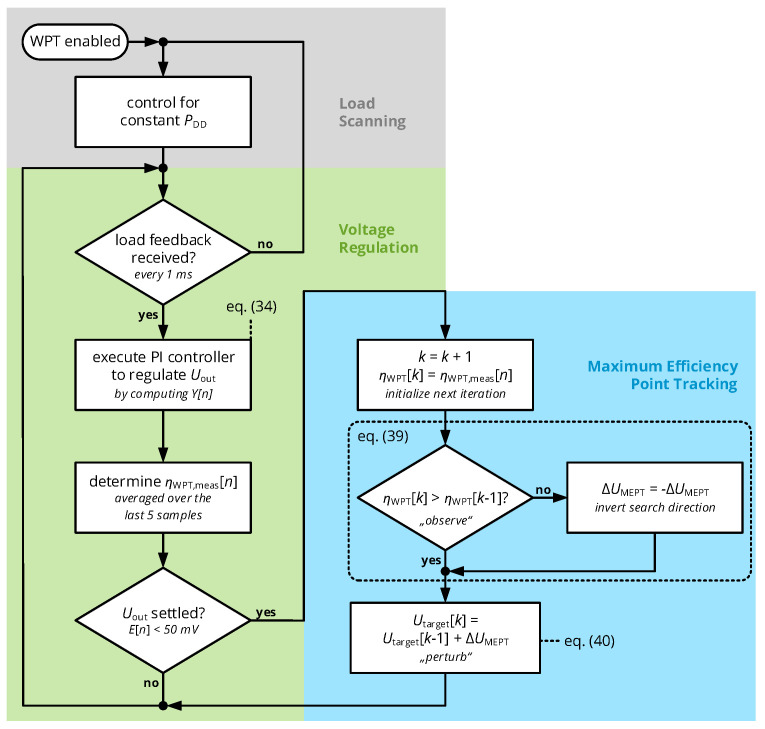
Flowchart of the algorithms for voltage regulation and maximum efficiency point tracking. The voltage controller is directly started after receiving a feedback signal from a wireless receiver. Once settled, the maximum efficiency point tracker is enabled, which modifies the setpoint of the voltage controller in the direction of increasing efficiency.

The nested mechanisms of power feedback (by voltage regulation) and maximum efficiency point tracking are graphically summarized in the flowchart of Figure 9.

## 4. System Realization

### 4.1. Hardware

**Figure 10:**
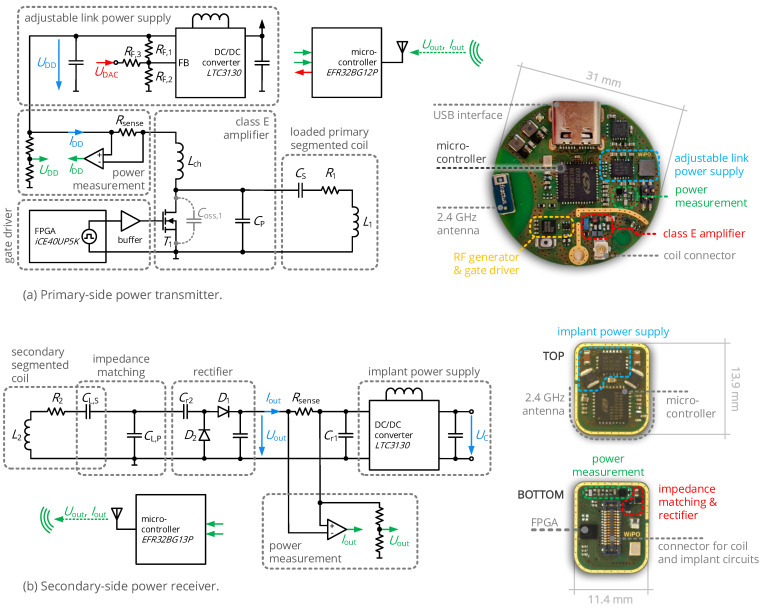
Schematics and PCB-level implementations of the power-conditioning circuits.

The power conditioning circuits for primary and secondary side were implemented as two miniaturized electronic devices based on printed-circuit-board (PCB) technology, shown in Figure 10. Both include a system on chip with a Cortex-M4F low-power microcontroller and 2.4 GHz radio interface (*Silicon Labs EFR32BG*) to execute measurement and control tasks. Moreover, both devices can measure the ingoing or outgoing DC power level at the WPT interface ($P_{\mathrm{DD}}$ and $P_{\mathrm{out}}$) by the combination of a shunt resistor and a *Maxim MAX9634* current-sense amplifier for current measurement and a voltage-divider configuration for voltage measurement; the microcontrollers’ analog-to-digital converters are used to sample their output. Further circuit components are selected according to Table 2. The capacitive series elements $C_{S}$ and $C_{L,S}$ were included into the coil traces (capacitive segmentation, cf. [8]) to limit dielectric losses.

In particular, the primary side features an adjustable voltage converter to realize the control of the rectifier output voltage: a *Linear Technology LTC3130* buck-boost converter with a *Murata LQH3NPZ* 10 µH inductor is complemented by a feedback resistor $R_{F3}$, through which an additional biasing voltage $U_{\mathrm{DAC}}$ (generated by the digital-to-analog converter of the microcontroller) is injected to modify $U_{\mathrm{DD}}$. A low-power field-programmable gate array (FPGA, a *Lattice iCE40UP*) generates the 40.68 MHz RF signal driving the gate of the class E amplifier’s transistor; a logic buffer integrated circuit (*Nexperia 74AUP2G16*) operates as a gate driver.

The secondary side applies a low-power buck-boost converter (*Linear Technology LTC3130* with a *Wuerth WE-PMI* 10 µH inductor) with wide input range, configured for a constant output voltage of $U_{C}=2.5\;V$ to supply the load circuits and the secondary-side microcontroller. As the power receiver board is designed as a small-scale circuit with a footprint of only 11.4 mm × 13.9 mm, a breakout board was designed to access analog and digital signals for system evaluation.

### 4.2. Software

With respect to software, the microcontroller of the secondary side samples rectifier output current and voltage ($U_{\mathrm{rect}}$ and $I_{\mathrm{rect}}$) by two consecutive measurements with the microcontroller’s ADC, triggered by a timer interrupt at a period of 1 ms. Subsequently, the measurement result is wirelessly transmitted to the primary side.

The primary side executes the algorithm defined in the flowchart of Figure 9: After system startup, the power amplifier is enabled with a constant supply voltage to emit a constant RF power level of approximately 500 mW. Once the secondary side is powered up by this initial startup sequence, it begins to transmit the momentary value of $U_{\mathrm{rect}}$ and $I_{\mathrm{rect}}$ as described before, so that the voltage controller algorithm can be executed: Here, the error term E[n] is computed as the difference of $U_{\mathrm{out}}\left[n\right]$ and the target voltage $U_{\mathrm{target}}\left[n\right]$ (initially set to $U_{\mathrm{target}}\left[0\right]=7\;V$) and substituted into (34) to compute the output of the PI controller Y[n] (with the dimension of a voltage), which is then applied to the DC/DC converter by the microcontroller’s digital-to-analog converter. For every execution step *n* of the voltage controller, primary-side input voltage $U_{\mathrm{DD}}$ and current $I_{\mathrm{DD}}$ are measured to compute the momentary system efficiency $U_{\mathrm{out}}\left[n\right]\cdot I_{\mathrm{out}}\left[n\right]/\left(U_{\mathrm{DD}}\left[n\right]\cdot I_{\mathrm{DD}}\left[n\right]\right)$, which is averaged over the last five samples to obtain the moving average of the total system efficiency $\eta_{\mathrm{WPT},\mathrm{meas}}$.

Whenever the voltage controller has settled, i.e., the voltage controller’s error term stays below 50 mV in three subsequent sampling steps, the maximum efficiency point tracking algorithm is executed (with the step index *k*): here, the total system efficiency $\eta_{\mathrm{WPT}}\left[k\right]=\eta_{\mathrm{WPT},\mathrm{meas}}$ is acquired and compared to the last sample $\eta_{\mathrm{WPT}}\left[k-1\right]$, in order to determine the future search direction of the target voltage according to the principles of the perturb-and-observe algorithm. Hence, Expressions (39) and (40) are evaluated to set the new value of the target voltage, which is further used by the voltage controller.

The parameters of the controllers are chosen as derived above and compiled in Table 3. The perturbance of the MEPT algorithm was configured to be $|\Delta U_{\mathrm{MEPT}}|=500\;\mathrm{mV}$.

In summary, the receiver only executes minimal measurement tasks, which can run in a lightweight task along any main application, while the computations for power feedback and maximum efficiency point tracking are offloaded on to the transmitter side.

## 5. Experimental Results

In the following section, the realized system is to subject to a series of static and dynamic measurements to evaluate the theoretical foundations of voltage control and efficiency optimization. All experiments implement the case scenario specified in Section 1.2, i.e., a brain implant with loosely coupled coils and a highly dynamic power consumption.

### 5.1. Efficiency vs. Rectifier Output Voltage

**Figure 11:**
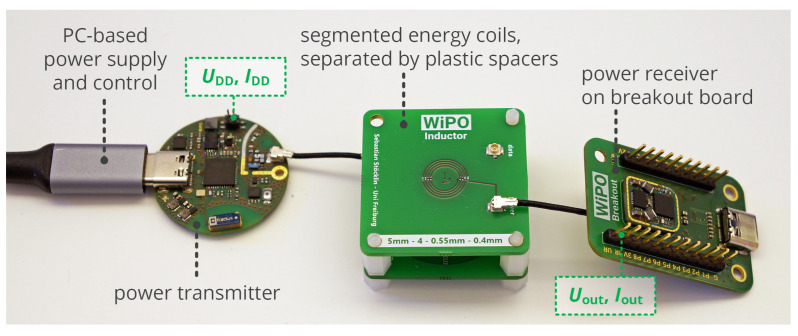
Measurement setup for the experimental analysis, including the analog test points for voltage and current measurement.

In the first step of the practical evaluation, the relation of rectifier output voltage and efficiency, being the essential relation required for the given efficiency point tracking mechanism, is analyzed. In this basic configuration, the power transfer coils are separated by a distance $d_{12}$ of 10 mm leading to $M_{12}=46\;\mathrm{nH}$. The primary-side supply voltage $U_{\mathrm{DD}}$ is swept for two constant output power levels $P_{C}$ of 30 mW and 90 mW, while DC input and output voltages and currents of the link ($U_{\mathrm{DD}}$, $I_{\mathrm{DD}}$, $U_{\mathrm{out}}$, $I_{\mathrm{out}}$) are acquired by a *Saleae Logic Pro 16* analyzer with the setup shown in Figure 11. From the measurements, the overall efficiency $\eta_{\mathrm{total}}=P_{\mathrm{out}}/P_{\mathrm{DD}}$ and the DC load resistance $R_{L,2}=U_{\mathrm{out}}/I_{\mathrm{out}}$ are computed.

**Figure 12:**
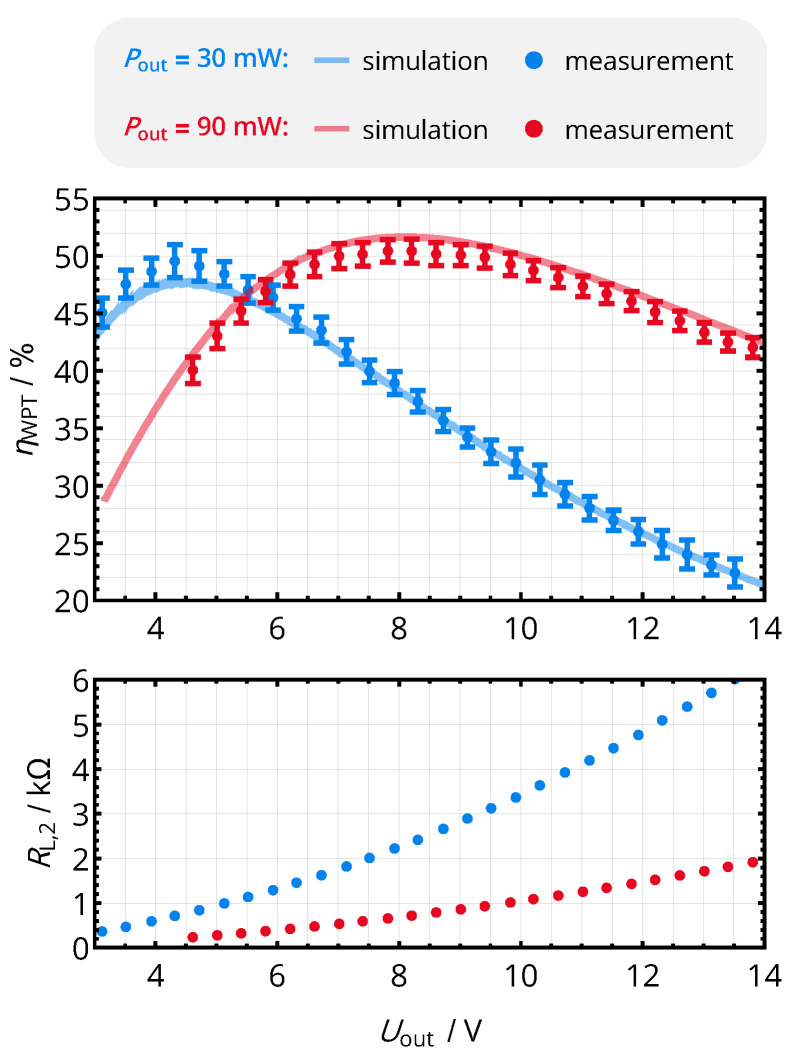
Overall system efficiency and effective DC load resistance versus the rectifier output voltage $U_{\mathrm{out}}$ for constant load power levels $P_{\mathrm{out}}$. The coil separation distance was set to $d_{12}=10\;\mathrm{mm}$ ($M_{12}=46\;\mathrm{nH}$). The simulation values are obtained from a harmonic balance simulation using *Keysight Advanced Design System 2016*.

The relation of the given parameters and the rectifier output voltage $U_{\mathrm{out}}$ is visualized in Figure 12: for a constant circuit load, $U_{\mathrm{out}}$ defines the load impedance and therefore the efficiency. As $R_{L,2}$ increases monotonically with $U_{\mathrm{out}}$, there is a dedicated and unique voltage that optimizes the overall efficiency, so that the system is suitable for the envisioned maximum efficiency point tracking mechanism.

The measured efficiency levels were also verified with the harmonic balance simulation setup described in Section 2.2. Parametric sweeps were performed for $U_{\mathrm{DD}}$ and $R_{L,2}$. The efficiency values with the appropriate combination of $U_{\mathrm{out}}$ and $P_{\mathrm{out}}$ were selected for the plot. A maximum relative error of 10% was acquired, so that the simulation setup is generally suitable to reproduce the behavior of the nonlinear power electronics and the 40.68 MHz RF coil system.

### 5.2. Voltage Controller for Power Feedback

The operability and stability of the voltage controller was analyzed for the maximum nominal coil separation distance of 20 mm, as the increased distance leads to a higher time constant $\tau_{\mathrm{WPT}}$ due to a higher Thévenin resistance. The maximum efficiency point tracking algorithm was deactivated to perform the measurements.

**Figure 13:**
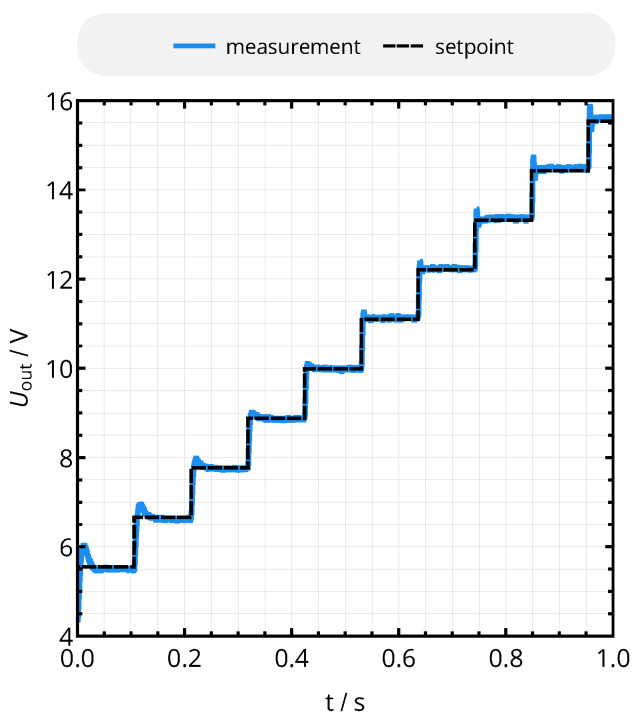
Series of step responses of the voltage control loop for $d_{12}=20\;\mathrm{mm}$ ($M_{12}=14\;\mathrm{nH}$) and $P_{\mathrm{out}}=25\;\mathrm{mW}$. Overshoots and rise time change with $U_{\mathrm{out}}$, as the latter determines the effective load resistance $R_{L,2}$ and, therefore, the characteristics of the transfer function.

Applying a series of steps in the setpoint of the controller across the suitable output voltage range results in the system behavior of Figure 13: it can be noted that reaction time and overshoots vary with the operating point (i.e., with the load resistance $R_{L,2}$), as theoretically predicted by (30) for the transfer function of the WPT interface. However, the system is stable across the considered voltage and load range.

**Figure 14:**
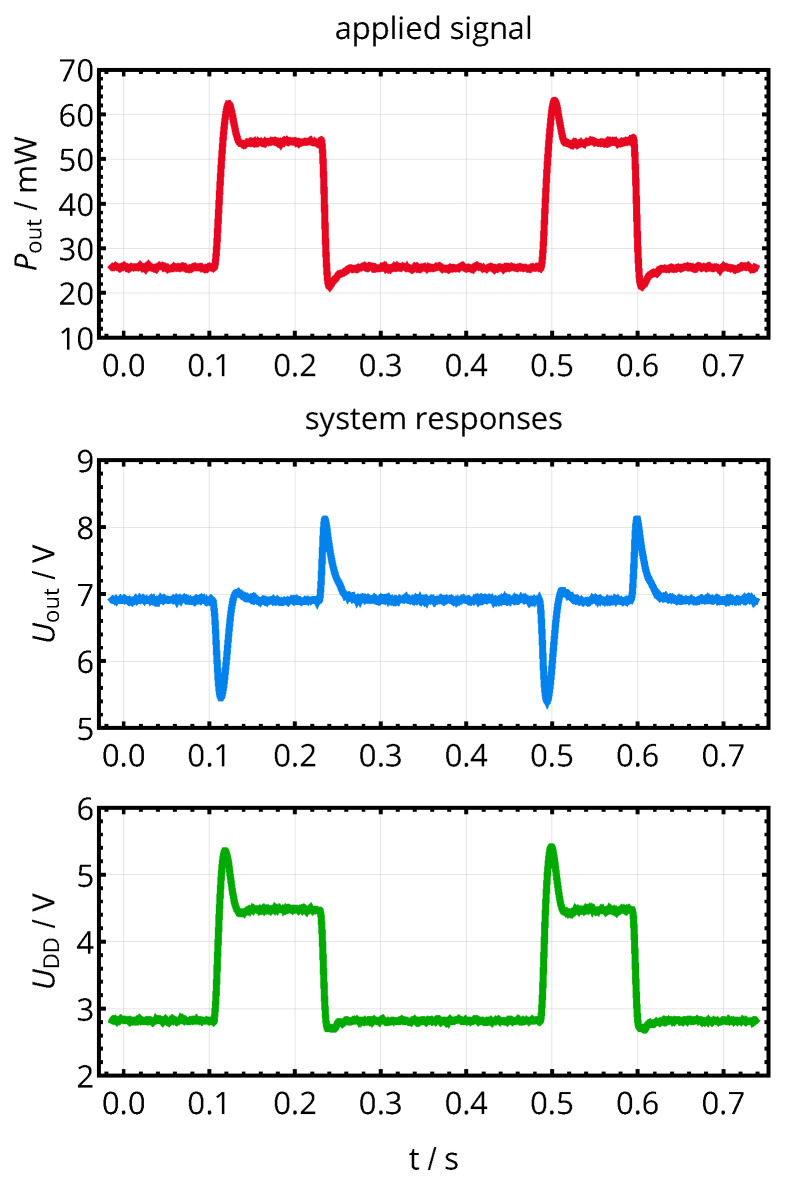
Reaction of the voltage controller with respect to $U_{\mathrm{out}}$ and $U_{\mathrm{DD}}$ due to a transient change in the load power consumption $P_{\mathrm{out}}$. For the given coil separation distance of $d_{12}=20\;\mathrm{mm}$ ($M_{12}=14\;\mathrm{nH}$), the target voltage of 7 V is reestablished within approximately 40 ms.

An exemplary reaction of the voltage controller to transient changes of the load power consumption is provided in Figure 14, where the power consumption $P_{\mathrm{out}}$ rises from the previous level of 25 to 55 mW. The controller reacts by increasing $U_{\mathrm{DD}}$ accordingly, so that $U_{\mathrm{out}}$ is reestablished to its preliminary value of 7 V within a time span of approximately 40 ms for the given configuration.

### 5.3. Maximum Efficiency Point Tracking

Finally, voltage controller and maximum efficiency point tracker are both enabled as provided in the flowchart of Figure 9, in order to evaluate their beneficial effect for system stability and efficiency.

First, the system is operated at the nominal operational distance of 10 mm with a base power consumption level of 25 mW. After 0.3 s of acquisition time, the power consumption is increased to 90 mW, and it is decreased back to 25 mW at 1.2 s. Input and output currents are recorded as described in Section 5.1. Additionally, the system efficiency levels acquired by the embedded system of the primary side (the basis for the decision-making of the MEPT circuit) at each execution step of the perturb-and-observe algorithm are extracted by a digital serial interface.

**Figure 15:**
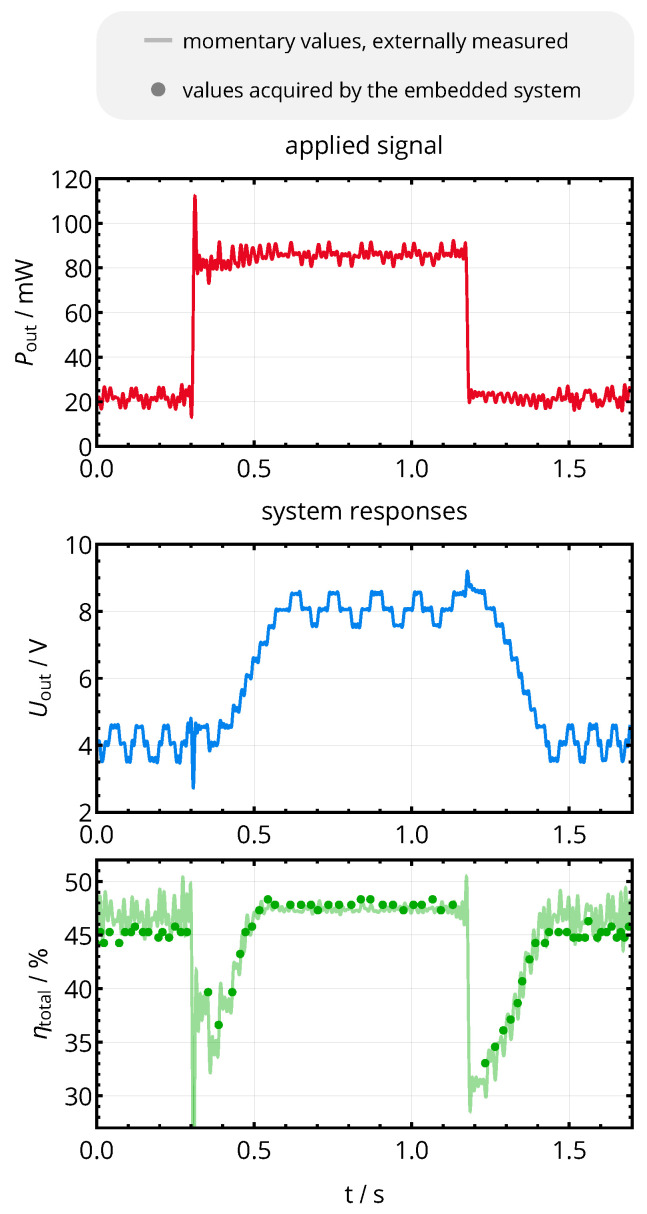
Reaction of the complete power feedback and maximum efficiency point tracking system with respect to a transient change in power consumption $P_{\mathrm{out}}$ for a coil separation distance of $d_{12}=10\;\mathrm{mm}$ ($M_{12}=46\;\mathrm{nH}$). The output voltage shows the characteristic behavior of the perturb-and-observe algorithm, oscillating around the optimal voltage for a constant output power level. Upon a load change, the system reestablishes the maximum efficiency level.

The system’s reaction with respect to output voltage $U_{\mathrm{out}}$ and overall efficiency $\eta_{\mathrm{total}}$ is shown in Figure 15: during the initial 0.3 s, the output voltage oscillates around the optimal voltage of approximately 4 V (the optimum voltage according to Figure 12), yielding an efficiency of approximately 47%. After the load change at 0.3 s, the output voltage exposes a large drop, which is counteracted by the voltage controller. The MEPT controller continues its operation just after reaching a stabilized voltage level at 0.35 s. It converges stepwise towards an output voltage of 8 V, being the optimal voltage for $P_{\mathrm{out}}=90\;\mathrm{mW}$ according to Figure 12. Again, $U_{\mathrm{out}}$ oscillates around the optimal value due to the nature of the perturb-and-observe algorithm. After the inverse load transient at 1.2 s, the overall efficiency can be reestablished from 30% back to 47% within a timespan of approximately 300 ms. Eventual short-term and noisy peaks in the efficiency curve of Figure 15 do not stem from a perturbance of the operating point, but just represent the charging behavior of $C_{r1}$ (cf. Figure 10), which is captured by the current sensor.

**Figure 16:**
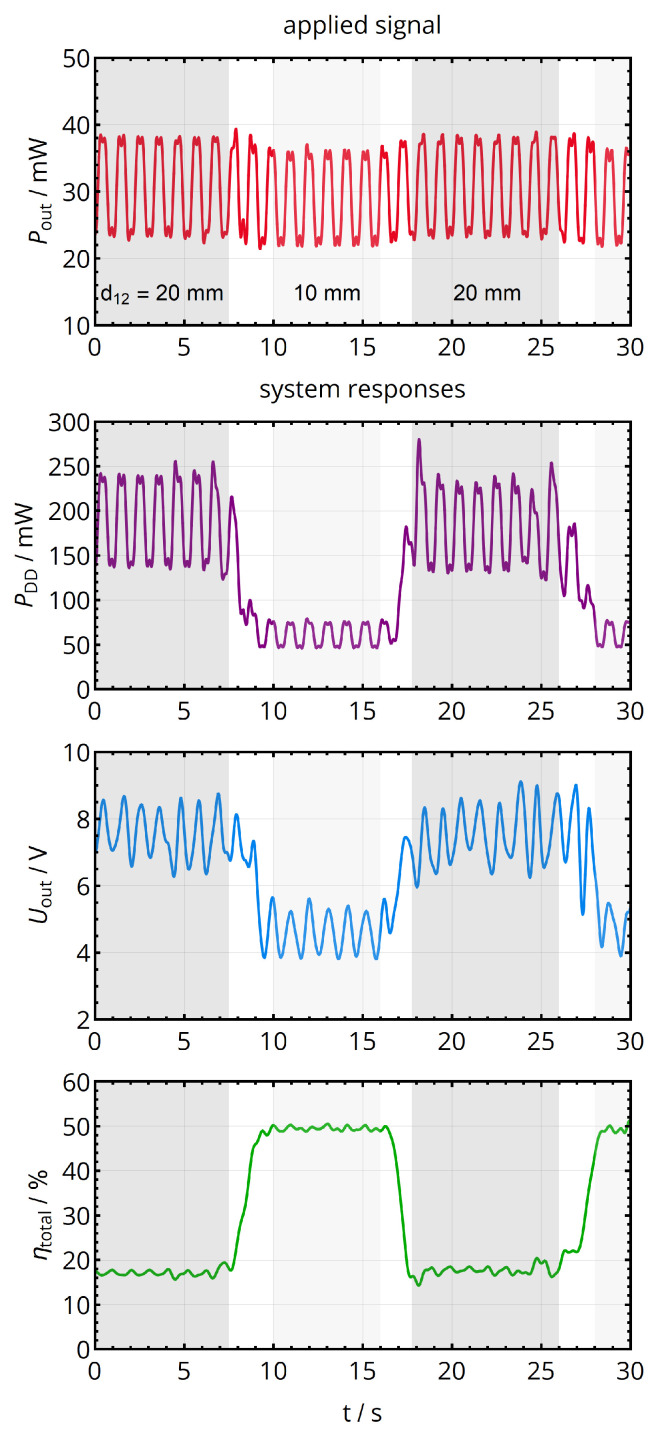
Reaction of the complete power feedback and maximum efficiency point tracking system with respect to a change in load power consumption (every 500 ms) and a change in coil separation distance (changing between 10 mm and 20 mm approximately every 10 s). The signals were low-pass filtered to show the time-averaged behavior without the individual switching events of the controllers. Both load and coil distance changes are automatically compensated.

In a last experiment on a larger time scale, the coil separation distance changes between 10 mm and 20 mm (i.e., $M_{12}$ changes between 46 nH and 14 nH) approximately every 10 s, while the power consumption was set to oscillate between 25 mW and 55 mW with a frequency of 1 Hz. The resulting time sequences of primary-side DC power $P_{\mathrm{DD}}$, the load voltage $U_{\mathrm{out}}$ and the efficiency $\eta_{\mathrm{total}}$ of Figure 16 were low-pass filtered with a cutoff frequency at 3 Hz to illustrate the time-averaged behavior without resolving the individual perturbances by the controllers. It can be seen that the output voltage oscillates with 1 Hz to maintain the maximum efficiency levels regardless of the power consumption (see the plateaus of $\eta_{\mathrm{total}}$ with only a small ripple). When changing the distance, the corresponding maximum efficiency levels of 20% and 50% are established by the adaption of the output voltage level.

### 5.4. Power Consumption of the RF and Control Circuits

While the efficiency of the wireless power transfer interface is the objective function of the optimization, the self-consumption of the primary- and secondary-side microcontroller systems should not exceed the energy saved due to the optimization. As a result, the power consumption of both devices was measured with respect to four main categories, as summarized in Table 4 and detailed as follows:

- Configuration: This includes the basic primary-side microcontroller circuit, which allows enabling and tuning the DC/DC converter, communicating with a PC-based control application, monitoring the temperature of the power amplifier, etc.
- RF signal generation: This summarizes the oscillator and the phase-locked loop to generate the RF signal as well as the gate driver interfacing the class E amplifier’s transistor. Together with the configuration circuits, these are the basic circuits required irrespective of any adaptive tuning to set up the WPT interface.
- Adaptive tuning includes the power overhead required to measure currents and voltages and to execute the controllers along a main task. Here, the additional energy consumption of timer and analog-to-digital converters as well as the energy consumption of the processor executing the controllers are registered. For the primary-side controller, the processing time shows a significant duration of 150 µs with a period of 1 ms, running along the main configuration task.
- Wireless communication: Here, the primary side is in permanent reception mode, while the secondary side sends packets including 4 bytes of measurement data every 1 ms. Two alternatives are outlined: the off-the-shelf 2.4 GHz transceiver integrated into the given wireless microcontroller (with an output power level of 0 dBm and a custom protocol with minimal overhead) versus the custom low-power near-field communication (NFC) system published by the authors of this work in [33]. The custom NFC system operates at a carrier frequency of 13.56 MHz and is therefore situated below the energy carrier of 40.68 MHz, so that no harmonics of the WPT interface fall into the communication band. Generally, please note that, by integrating the data communication into any regular data stream, which is set up independently of the efficiency optimization to transmit sensor information, the effective energy overhead with respect to wireless communication can be strongly reduced.

## 6. Discussion

Both the theoretical analysis and the experimental evaluation of the adaptive tuning mechanisms resulted in a series of concepts and findings that enable a small-scale and low-power wireless power transfer interface with favorable stability and efficiency metrics:

First, low-power inductive links practically do not need an **adaptive output network** or adaptive tuning of the operational frequency when operating with coils of high quality factor ($Q_{i}>50$) and when disposing at least some parallel-resonant compensation in the output matching network (α≤0.6). This strongly simplifies the design and allows to stay within the required ISM bands for operation of inductive links with relatively high frequencies.

Moreover, the distribution of reactance between the series element $X_{L,S}$ and the parallel element $X_{L,P}$, indicated by the compensation ratio α, allows **selecting the power band** in which optimal matching is achieved across the required range of mutual inductances. This finding also implies that the voltage range of the rectifier and the load-side DC/DC converter limit the tuning range of adaptive impedance matching mechanisms—the larger is the voltage range, the larger is the range of power levels to be tuned to the optimal resistance.

While Section 2.2 provides insight into how to **simulate the complete radio-frequency chain of the wireless power transfer link** and how to extract the efficiency impact of individual components, it also shows a series of circuit properties: Firstly, Greinacher rectifiers with Schottky diodes can operate as very efficient AC/DC circuits, reaching an efficiency of more than 90% at operational frequencies in the range of 40.68 MHz. Secondly, load resistances optimizing the efficiency of the complete link do not necessarily fall together with those optimizing the efficiency of the coil system. Moreover, they are dependent on the power level, such that efficiency-optimization approaches based on transfer functions (relying on the linearity of all devices) do not work for high-frequency systems with strongly nonlinear power electronics. However, the overall efficiency has a particular optimum, so that it can be optimized even by relatively simple approaches such as perturb-and-observe algorithms.

Assuming a constant voltage transfer function of these nonlinear devices (with voltage gains of $k_{\mathrm{amp}}$ and $k_{\mathrm{rect}}$), we could obtain a relatively compact and simple PT1 transfer function of the complete wireless power transfer interface with an analytical approach. This allows designing a digital controller for the **rectifier’s output voltage regulation** in z-domain, which was also stable in the experimental evaluation. However, combining the model with the effects of the DC/DC-converter load, for which the load resistance is a function of the output voltage such as given by (38), and, noting that the Thévenin resistance of (29) is also a function of the load resistance $R_{L,2}$, the system is actually nonlinear, so that the linear stability analysis for various combinations of ($M_{12}$, $R_{L,2}$) are technically only valid for small perturbations of the output voltage $U_{\mathrm{out}}$. Even though the realized system is able to compensate transient changes in power consumption and mutual inductance, these must be classified as transient changes in the resistances and reactances of the interface, so that the system becomes a time-varying system. This identifies a numerical simulation of this nonlinear and time-varying system as a central request for future work.

The particular advantages of the presented **maximum efficiency point tracking concept** perturbing the setpoint of the voltage controller are that:

- It operates with a simple topology at the secondary side, using a standard output-regulated DC/DC converter at the load and requiring only a measurement of the DC output voltage and current.
- Its algorithm can easily be incorporated into the primary-side digital control unit.
- It can act on top of the voltage controller, which assures system stability. This is even improved by triggering the MEPT algorithm only when the error term of the voltage controller $|U_{\mathrm{out}}-U_{\mathrm{target}}|$ has decreased below a certain threshold.

In the practical evaluation, the tracking system successfully reestablished the maximum efficiency point: With achieved efficiency levels of around 50% as well as optimal voltages of 4 V for 25 mW and 8 V for 90 mW, as shown in Figure 15, the operating points established by the MEPT algorithm correspond to the optimal values obtained in the analysis of Figure 12.

With respect to the self-consumption of the power-conditioning circuits, the **overall power budget** of the adaptive impedance matching solution is important to be evaluated: the power overhead due to adaptive matching includes the positions of adaptive tuning and wireless communication of Table 4, which results in an increase of the overall power consumption by

(41) $$\begin{array}{c} \Delta P_{\mathrm{DD},\mathrm{overhead}}=P_{1,\mathrm{overhead}}+\frac{P_{2,\mathrm{overhead}}}{\eta_{\max}}. \end{array}$$

with the custom NFC technology, voltage regulation and MEPT could be realized with a power overhead $P_{1,\mathrm{overhead}}\approx 7.6\;\mathrm{mW}$ on the primary side and a self-consumption of $P_{2,\mathrm{overhead}}\approx 1\;\mathrm{mW}$ at the secondary side, which yields a total power overhead of $\Delta P_{\mathrm{DD},\mathrm{overhead}}\approx 9.7\;\mathrm{mW}$ at a separation distance of $d_{12}=10\;\mathrm{mm}$ ($\eta_{\max}\approx 47\%$).

If the efficiency increase outweighs this additional power requirement must be evaluated for the individual application. While a regular system would experience a degraded efficiency $\eta_{2}$ when switching from a power level $P_{\mathrm{out},1}$ with optimal matching to a second power level $P_{\mathrm{out},2}$, the adaptive system can reestablish the efficiency $\eta_{\max}$. This will result in a power benefit

(42) $$\begin{array}{c} \Delta P_{\mathrm{DD},\mathrm{benefit}}=P_{\mathrm{out},2}\cdot \frac{t_{2}}{t_{1}+t_{2}}\cdot \left(\frac{1}{\eta_{2}}-\frac{1}{\eta_{\max}}\right), \end{array}$$

where $t_{i}$ is the time span during which the power level $P_{\mathrm{out},i}$ is applied. For the example of Figure 15, reestablishing the efficiency from 38% to 47% after switching to $P_{\mathrm{out},2}=90\;\mathrm{mW}$ would cause a power benefit of $\Delta P_{\mathrm{DD},\mathrm{benefit}}\approx 23\;\mathrm{mW}$ (for $t_{1}=t_{2}$), so that the overhead of 9.7 mW with the custom NFC system is feasible. With the 2.4 GHz communication interface, we obtain $\Delta P_{\mathrm{DD},\mathrm{overhead}}\approx 51\;\mathrm{mW}$, which is larger than the benefit even when considering $t_{2}\gg t_{1}$ ($\Delta P_{\mathrm{DD},\mathrm{benefit}}\approx 43\;\mathrm{mW}$). This result indicates that it is vital to use a low-power communication technology.

However, Equation (42) assumes that the link was optimally configured for the state of $P_{\mathrm{out},1}$, which is not necessarily given without an MEPT system, so that the practical benefit of adaptive tuning will most likely be higher. Moreover, it must also be considered that the given adaptive power overhead $\Delta P_{\mathrm{DD},\mathrm{overhead}}$ is not just due to the efficiency optimization, but also due to the voltage regulation for power feedback, which is highly beneficial for critical applications such as biomedical sensor systems.

The **time to reestablish the maximum efficiency point** of approximately 300 ms identifies which type of transient changes can be compensated: for power changes of longer duration, adaptive matching is possible; for shorter duration of the individual power consumption levels, the optimum will not be reached. For the given case scenario of a neural implant, time constants of physiological changes and periods stimulation are in the range of a few 100 ms to 1 s [34], so that the exemplary implementation would allow for the maximization of the efficiency in these types of biomedical systems.

The **additionally required electric components** to implement our concepts include a current sense amplifier, a two-channel analog-to-digital converter as well as a wireless communication interface for both primary and secondary side. While typical primary-side reader units are equipped with these components anyhow, hardware components and cost of an adaptive receiver do almost double compared to a simple receiver topology only including matching network, rectifier and voltage regulator. For the case of active biomedical sensor systems already featuring a secondary-side microcontroller and a wireless interface, the concept can be implemented with a minor increase of cost due to the additional current sense amplifier.

**Figure 17:**
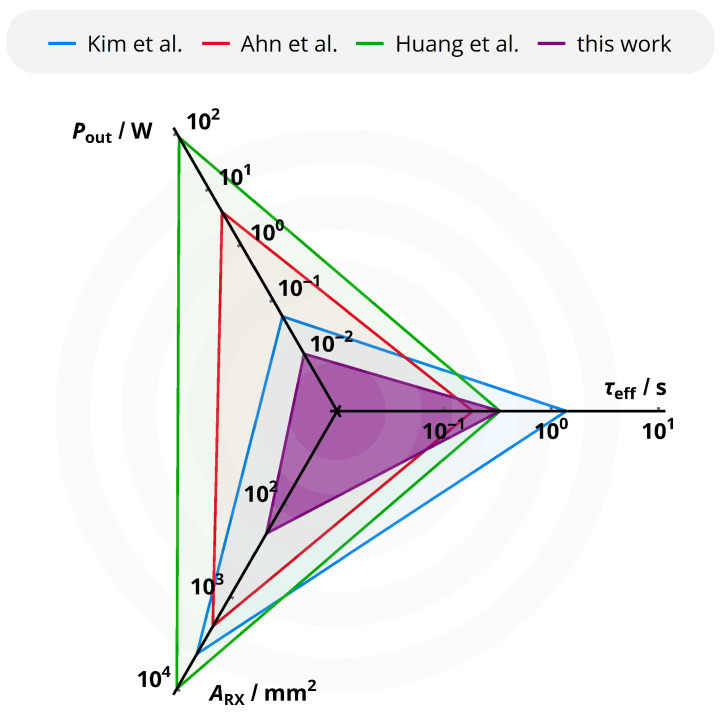
Main characteristics of different maximum efficiency point tracking systems reported in the literature: typical output power level $P_{\mathrm{out}}$, receiver circuit footprint $A_{\mathrm{RX}}$ and typical time to reestablish the maximum efficiency $\tau_{\mathrm{eff}}$. For all metrics, smaller values are better to accomplish the objectives of low-power biomedical sensor systems.

For the practical application of an adaptive WPT impedance matching system, a series of characteristics are of major importance: First, the power levels $P_{\mathrm{out}}$ for which adaptive matching is possible determines the category of devices the system is suitable for. This also holds for the circuit size, especially for the PCB footprint $A_{\mathrm{RX}}$ of the receiver circuit. Lastly, the time $\tau_{\mathrm{eff}}$ required to reestablish the optimal efficiency is crucial for the dynamic behavior of the system. These quantities are provided in Table 5 for a set of **maximum efficiency point tracking systems reported in the literature** and compared to the concept of this paper in the radar plot of Figure 17. In summary, the given system features the lowest power levels and the smallest receiver size, while being on par with the systems disposing the fastest reaction times. As a result, the combination of the rectifier output voltage regulation with a perturb-and-observe-based MEPT tracker is particularly suitable for low-power and small-size wireless power transfer systems such as required by modern biomedical sensor systems.

## Figures and Tables

**Table 1:** Dimensions and characteristics of the used WPT coils, including outer radius $r_{i}$, number of turns $N_{i}$, trace width $w_{i}$ and trace pitch $p_{i}$. The effective equivalent circuit parameters are provided for f=40.68MHz, while maintaining the coil current uniform by capacitive segmentation [8].

coil no. *i*	$r_{i}$	$N_{i}$	$w_{i}$	$p_{i}$	$L_{i}$	$R_{i}$	$M_{12}\left(d_{12}\right)$
1	15 mm	10	0.4 mm	0.5 mm	3710 nH	4.72 Ω	5 mm: 82 nH 10 mm: 46 nH 20 mm: 14 nH
2	5 mm	4	0.4 mm	0.5 mm	190 nH	0.84 Ω	

**Table 2:** Components and component values of the applied RF power electronics. $C_{L,P}$ is reduced by 20 pF from the value in Section 2.1 to compensate for the input capacitance of the rectifier.

$L_{\mathrm{ch}}$	$T_{1}$	$C_{P}$	$C_{S}$	$C_{L,S}$	$C_{L,P}$	$C_{r2}$	$C_{r1}$	$D_{1}$, $D_{2}$
2.2 µH	*EPC8002*	40 pF	4.2 pF	160 pF	140 pF	100 nF	16 µF	*BAT54LPS*

**Table 3:** Parameters and output quantities of the voltage controller and the MEPT algorithms.

Output Voltage Controller						Maximum Efficiency Point Tracker	
$T_{S}$	$K_{P}$	$T_{I}$	$T_{D}$	$U_{\mathrm{target}}\left[0\right]$	Y[n]	$|\Delta U_{\mathrm{MEPT}}|$	$U_{\mathrm{target}}\left[k\right]$
1 ms	0.07	0.01 s	0 s	7 V	see (34)	500 mV	see (39) and (40)

**Table 4:** Self-consumptions of the custom wireless power transfer circuits.

Sub-Circuit	Energy Transmitter		Energy Receiver	
configuration	16.4 mW		—	
RF signal generation	18.0 mW		—	
adaptive tuning	5.1 mW ^1,2^		925 µW ^1^	
wireless communication	2.4 GHz	custom NFC	2.4 GHz	custom NFC
	33.0 mW	2.5 mW	5080 µW	53 µW ^3^
**tuning overhead ^4^**	**38.1 mW**	**7.6 mW**	**6005 µW**	**978 µW**
**total**	**72.5 mW**	**42.0 mW**	—	—

^1^ overhead of the voltage and current measurements along a main task; ^2^ overhead of the controllers’ computations; ^3^ with a packet duration of 12 μs, transmitted every 1 ms; ^4^ sum of adaptive tuning and wireless communication.

**Table 5:** Main characteristics of maximum efficiency point tracking systems reported in the literature.

Author	Tuning	$P_{\mathrm{out}}$	$f_{0}$	$A_{\mathrm{RX}}$	$\tau_{\mathrm{power}}$	$\tau_{\mathrm{eff}}$	Ref.
Kim et al.	parallel	45 mW	13.56 MHz	3500 mm^2^	—	1.2 s	[16]
Zhong et al.	series	2.5 W	100 kHz	N/A	-	20 s	[19]
Ahn et al.	series	3–10 W	305 kHz	1800 mm^2^	0.17 s	0.17 s	[21]
Yeo et al.	series	5 W	6.78 MHz	880 mm^2^	-	-	[20]
Huang et al.	series	60 W	200 kHz	8100 mm^2^	0.3 s	0.3 s	[22]
**this work**	**L network**	**10–100 mW**	**40.68 MHz**	**192 mm** ${}^{2}$	**0.1 s**	**0.3 s**	—

## Data Availability

The raw data of graphs and tables presented in this study are available on request from the corresponding author.

## Abbreviations

The following abbreviations are used in this manuscript:

AC	Alternating Current
DC	Direct Current
ISM	Industrial, Scientific and Medical (frequency bands)
MEPT	Maximum Efficiency Point Tracking
NFC	Near Field Communication
PCB	Printed Circuit Board
PID	Proportional-Integral-Derivative (controller)
RF	Radio Frequency
WPT	Wireless Power Transfer
